# Individual level covariate adjusted conditional autoregressive (indiCAR) model for disease mapping

**DOI:** 10.1186/s12942-016-0055-7

**Published:** 2016-07-29

**Authors:** Md. Hamidul Huque, Craig Anderson, Richard Walton, Louise Ryan

**Affiliations:** 1 School of Mathematical and Physical Sciences, University of Technology Sydney, 15 Broadway, Ultimo, NSW 2007 Australia; 2 Australian Research Council Centre of Excellence for Mathematical and Statistical Frontiers, ​Parkville, VIC, 3010 Australia; 3 Cancer Institute NSW, 8 Central Avenue, Eveleigh, NSW 2015 Australia

**Keywords:** Covariate adjustment, Disease mapping, Geographical variation, Neutropenia, Spatial model

## Abstract

**Background:**

Mapping disease rates over a region provides a visual illustration of underlying geographical variation of the disease and can be useful to generate new hypotheses on the disease aetiology. However, methods to fit the popular and widely used conditional autoregressive (CAR) models for disease mapping are not feasible in many applications due to memory constraints, particularly when the sample size is large. We propose a new algorithm to fit a CAR model that can accommodate both individual and group level covariates while adjusting for spatial correlation in the disease rates, termed indiCAR. Our method scales well and works in very large datasets where other methods fail.

**Results:**

We evaluate the performance of the indiCAR method through simulation studies. Our simulation results indicate that the indiCAR provides reliable estimates of all the regression and random effect parameters. We also apply indiCAR to the analysis of data on neutropenia admissions in New South Wales (NSW), Australia. Our analyses reveal that lower rates of neutropenia admissions are significantly associated with individual level predictors including higher age, male gender, residence in an outer regional area and a group level predictor of social disadvantage, the socio-economic index for areas. A large value for the spatial dependence parameter is estimated after adjusting for individual and area level covariates. This suggests the presence of important variation in the management of cancer patients across NSW.

**Conclusions:**

Incorporating individual covariate data in disease mapping studies improves the estimation of fixed and random effect parameters by utilizing information from multiple sources. Health registries routinely collect individual and area level information and thus could benefit by using indiCAR for mapping disease rates. Moreover, the natural applicability of indiCAR in a distributed computing framework enhances its application in the Big Data domain with a large number of individual/group level covariates. CI NSW Study Reference Number: 2012/07/410. Dated: July 2012.

## Background

The risks of many diseases and health outcomes may vary across geographical locations because of locally varying distributions of socioeconomic, behavioural and environmental risk factors [1]. These spatially correlated risk factors can have important implications for the observed disease rates in small areas. Mapping disease rates over a region offers a visual illustration of geographical variation. These maps are particularly useful for generating new hypotheses through identifying apparently high risk areas or disease clusters [2]. However, producing such maps is complicated by the fact that raw incidence rates are often unstable due to small incidence counts, spatial correlation among rates and also due to the variation in individual patient characteristics [3–5].

Poisson mixed models with conditional autoregressive random effects are commonly used for assessing the relationship between a rare disease outcome and risk factors in the presence of geographical variation [6]. These models can adjust for region specific spatial random effects for correlated disease rates and both individual- and region-specific covariates. However, the fitting of such models is subject to high computational burden, particularly when the sample size is large and when the number of individual and group level covariates are large. To alleviate such problems, investigators often adjust for the age and sex distribution of the underlying population through calculation of an offset in the model [7]. Therefore, the effect of age and sex on disease risk can not be estimated from these models. Moreover, such an approach ignores a large number of potential individual level covariates that may be related to the underlying disease process and readily available in health registries.

Health registries routinely collect geo-coded information relating to the patient’s residence at diagnosis, their socio-demographic status and their clinical characteristics. In addition, information on locally varying socioeconomic, behavioral and environmental risk factors for each area under study can also be obtained from other data sources. For example, in Australia, New South Wales (NSW) cancer registries collect cancer treatment and outcome information for each patient diagnosed with cancer, along with their socio-demographic characteristics. Additionally, a socio-economic index for areas (SEIFA) and an area specific index for remoteness (ARIA) of each patient’s residence can be obtained from the Census Bureau. Combining these individual and area level characteristics in mapping studies can help researchers and policy makers to understand the relative contribution of both individual and group level covariates to the observed cancer rates. In addition, combining such data can also reduce ecological bias, which occurs when the group level exposure–disease relationship does not reflect the individual level relationship. A reduction in this bias leads to improved inference about both our group and individual level covariates [8, 9]. In this paper we propose a novel approach that enables the study of individual level risk factors in mapping studies.

The aim of our current research is to make use of routinely collected administrative cancer treatment and outcome data to explore the possible geographical variation in the rate of neutropenia admissions corresponding to all cancer types across NSW. Neutropenia is a blood disorder with an abnormally low number of neutrophil granulocytes (a type of white blood cell in the blood), often associated with fever. It is a life threatening complication of cancer chemotherapy and a major cause of morbidity and associated healthcare resource costs. Furthermore, neutropenia results in compromised efficacy due to delays and dose reductions in chemotherapy [10].

NSW is the most populated state in Australia with a population of approximately 7.6 million people. Geographical variations in neutropenia admissions are of particular interest because of the uneven geographical concentration of the population within the state. As a result of this uneven population density, the level of access to health care services is not uniform across the whole region [11]. Moreover, neutropenia incidence might also depend on patient age and cancer type, as treatment modalities often vary across different types of cancer and age groups. Therefore, appropriate analysis of geographical variation of neutropenia admissions requires adjustment for both the patient’s demographic characteristics and covariates reflecting the patient’s geographic location of residence. In our current application, we explore whether there is any spatial variation in the rates of neutropenia admissions after adjusting for patients’ individual and clinical characteristics.

In our proposed method, hereafter known as indiCAR, we incorporate individual level covariate information in a two step iterative procedure following an initialization step. At the initialization step, individual level outcome data were fitted against individual level covariates with a Poisson generalized linear model (GLM), ignoring random effects and group level covariates. Then, at the first step, the individual level outcome data were aggregated at the area level and fitted via a Poisson generalized linear mixed model (GLMM) against area level covariates including a conditional autoregressive spatial random effect, and an offset calculated based on individual covariate contributions. At the second step, the individual level outcome data is fitted via a Poisson GLM with individual level covariates and a second offset calculated based on the contribution of area specific covariates and random effects obtained from the previous step. Steps 1 and 2 are repeated until convergence.

We evaluate the performance of our indiCAR method through simulation studies and also compare indiCAR to the traditional method of age-sex standardisation [7]. Our simulation results show that the proposed indiCAR approach is able to correctly estimate coefficients associated with both individual and group-level covariates. Simulation studies also reveal that our approach is faster than existing approaches such as *hlmer* with CAR for fitting spatial random effects when the number of individuals within a group is low, and works for large sample sizes where these other methods fail. We illustrate our proposed indiCAR method using data on neutropenia admissions from the NSW Cancer Institute and conclude with some practical guidelines.

## Methods

### Data

NSW cancer registries were used to identify patients diagnosed with cancer, associated treatment procedures and co-morbidities. Specifically, we used data from the NSW Central Cancer Registry (CCR) linked to NSW Admitted Patient Data Collection (APDC). Detailed descriptions of the data items can be obtained from the Centre for Health Record Linkage (CHeReL http://www.cherel.org.au/master-linkage-key). Data were checked for consistency across data sources and linked by assigning a unique project person number (PPN) to each patient. Our study population comprises all cancer patients that were diagnosed with cancer and were hospitalized during the period between 2001 and 2009.

Demographic variables including age at diagnosis, gender, residence at diagnosis, postal area of residence, and the ARIA were obtained from the CCR database. The ARIA variable was recorded at individual level rather than postal area level because the ARIA index varies within postal areas. The SEIFA (an index of social disadvantage) and the geo-coded shape files for mapping corresponding to 2006 census postal areas were obtained from the Australian Bureau of Statistics (ABS). Individual level clinical characteristics such as type of cancer were also obtained from the CCR. The diagnosis of neutropenia admissions and co-morbidity were obtained using data from the APDC. The ICD-10-AM (International Statistical Classification of Disease and Related Health problem, 10th revision, Australian modification) code D70 (agranulocytosis) was used to identify admissions with possible neutropenia.

### The model

Suppose the total area under study is divided into *M* contiguous regions and the number of neutropenia admissions for the *ith* ($i=1,2,\ldots ,n_j$) individual in the *jth*$(j=1,2,\ldots ,M)$ region is denoted by $\{y_{ij}\}$. Let ${{\varvec{Y}}}$ be a vector with elements $\{y_{ij}\}$ that represents the number of neutropenia admissions for all individuals in the study regions of interest. Similarly, let ${\varvec{X}}=(X_1,X_2,\ldots ,X_p)$ and ${\varvec{U}}=(U_1,U_2,\ldots ,U_q)$ represent individual and area level covariate matrices with dimensions $n\times p$ and $M\times q$, respectively, where *n* is the total sample size i.e., $n=\sum \nolimits _{j=1}^M n_j$. We define a replication matrix, ${\varvec{Z}}$ of dimension ${n\times M}$ to map group level covariates and random effects to the individual level as

$$\begin{aligned} {\varvec{Z}}=\left[ \begin{array}{cccc} {\varvec{1}}_{n_1\times 1} &{}\quad {\varvec{0}}_{n_1\times 1} &{}\quad \cdots &{}\quad {\varvec{0}}_{n_1\times 1}\\ {\varvec{0}}_{n_2\times 1} &{}\quad {\varvec{1}}_{n_2\times 1} &{}\quad \cdots &{}\quad {\varvec{0}}_{n_2\times 1} \\ \vdots &{}\quad \vdots &{}\quad \ddots &{}\quad \vdots \\ {\varvec{0}}_{n_M\times 1} &{}\quad {\varvec{0}}_{n_M\times 1} &{}\quad \cdots &{}\quad {\varvec{1}}_{n_M\times 1} \end{array} \right] . \end{aligned}$$

Under the above specifications, conditional on the area specific random effect vector, ${\varvec{b}}$, the number of neutropenia admissions for each cancer patient is assumed to be a Poisson random variable with mean ${\varvec{\mu}}$, given by

1 $$\ln ({\varvec{\mu }})={\varvec{X}}{\varvec{\beta}}+{\varvec{Z}}{\varvec{U}}{\varvec{\gamma }}+{\varvec{Z}}{\varvec{b}}.$$

where ${\varvec{\beta}}$ and ${\varvec{\gamma }}$ are the vectors of regression coefficients associated with the individual level and group level covariates, respectively. Of course, it is possible to express model (1) by replicating group level covariate data to the individual level and including them within the design matrix, ${\varvec{X}}.$ However, such a formulation often results in high computational burden and a large amount of storage memory allocation. Instead, formulation (1) helps to fit individual and group level data separately in a distributed computing framework as will be shown at the end of the current section.

Many different choices for modelling the random effect, ${\varvec{b}}$ are available in the mapping literature (see [6], for a recent review). Among these, the method of Leroux et al. [7] is appealing because it allows varying weights between spatially structured and unstructured variation [7]. Within this framework, the random effect vector, ${\varvec{b}}$ has a multivariate normal distribution with mean ${\varvec{0}}$ and a covariance matrix, ${\varvec{D}}$ delivered through its Moore-Penrose generalized inverse, ${\varvec{D}}^{-}=\sigma ^{-2}\{(1-{\varvec{\lambda }})\varvec{I}+\lambda {\varvec{R}}\}$, where $\varvec{I}$ is the identity matrix, ${\varvec{R}}$ is the intrinsic auto regression matrix reflecting the neighbourhood structure. Typically, neighbours are those areas which share a common boundary, but distance based neighbourhood structures can also be used [12]. Underlying the Leroux et al. [7] approach is the specification of the generalized inverse of the covariance matrix ${\varvec{D}}$. This formulation therefore avoids inverting the covariance matrix ${\varvec{D}}$. Alternatively, one can restrict ${\varvec{\lambda }}$ to the range (0, 1), thus ensuring that ${\varvec{D}}$ is invertible. The typical element of ${\varvec{R}}$ is given by

$$\begin{aligned} {\varvec{R}}_{jj^{\prime }} =\left\{ \begin{array}{ll} m_j, &{}\quad j=j^{\prime } \\ -I\left\{ j\sim j^{\prime }\right\} &{}\quad j\ne j^{\prime }, \end{array}\right. \end{aligned}$$

where $m_j$ is the number of neighbours of region *j*, and $I\{j\sim j^{\prime }\}$ is an indicator function that takes value 1 if regions *j* and $j^{\prime }$ are neighbours and 0 otherwise. The parameters characterising the random effect distribution, ${\varvec{\theta }}= (\sigma ^2>0,{\varvec{\lambda }}\in [0,1])$ quantify overdispersion and spatial dependence respectively. A larger value of $\lambda \in [0,1]$ indicates a higher degree of spatial correlation among proximal areal units. This specification results in two extreme cases: (1) completely independent random effects when ${\varvec{\lambda }}=0$ and (2) the intrinsic autoregressive model when ${\varvec{\lambda }}=1$ [4]. In cases where $0< {\varvec{\lambda }}< 1$, a weighted combination of these extreme cases is assumed.

Since the random effects, ${\varvec{b}}$ are unobserved, inference about ${\varvec{\beta}}$, ${\varvec{\gamma }}$ and ${\varvec{\theta }}$ can be made by integrating out the distribution of the random effects, ${\varvec{b}}$. The corresponding integrated quasi-likelihood function is equal to (see equation (2) of Breslow and Clayton [13])

$$\begin{aligned} |{\varvec{D}}|^{-\frac{1}{2}}\int \exp \left[ -\frac{1}{2}\sum _{j=1}^M\sum _{i=1}^{n_j} d_{ij}(Y_{ij},\mu _{ij})-\frac{1}{2} {\varvec{b}}^{\mathrm{T}}{\varvec{D}}^{-}{\varvec{b}}\right] d{\varvec{b}}, \end{aligned}$$

where $d(Y,{\varvec{\mu }})$ refers to the deviance residual associated with observation *Y*.

The maximum likelihood estimates of ${\varvec{\beta}}$, ${\varvec{\gamma }}$ and ${\varvec{\theta }}$ are simply those values which maximize the above quasi-likelihood. However, no simple closed form expression exists for the integral. Instead, Breslow and Clayton [13] proposed the penalized quasi-likelihood (PQL) approach for parameter estimation and inference. The PQL uses Laplace’s method for integral approximation and jointly maximizes the following quasi-likelihood function to obtain estimates for ${\varvec{\beta}}$, ${\varvec{\gamma }}$ and ${\varvec{b}}({\varvec{\theta }})$ (see equation (6) of Breslow and Clayton [13])

2 $$-\frac{1}{2}\sum _{j=1}^M\sum _{i=1}^{n_j} d_{ij}(Y_{ij},\mu _{ij})-\frac{1}{2} {\varvec{b}}^{\mathrm{T}}{\varvec{D}}^{-}{\varvec{b}}.$$

Under the above specification the approximate log-likelihood can be expressed as

3 $$\begin{aligned}&const+{\varvec{Y}}^{\mathrm{T}}({\varvec{X}}{\varvec{\beta}}+{\varvec{Z}}{\varvec{U}}{\varvec{\gamma }}+{\varvec{Z}}{\varvec{b}})-{\varvec{1}}^{\mathrm{T}}\exp ({\varvec{X}}{\varvec{\beta}}+{\varvec{Z}}{\varvec{U}}{\varvec{\gamma }}+{\varvec{Z}}{\varvec{b}})-\frac{1}{2} {\varvec{b}}^{\mathrm{T}}{\varvec{D}}^{-}{\varvec{b}}. \end{aligned}$$

Differentiating (3) with respect to ${\varvec{\beta}}$, ${\varvec{\gamma }}$ and ${\varvec{b}}$ using vector matrix calculus [14], we obtain the following score equations

4 $$\left\{{\varvec{Y}}-\exp ({\varvec{X}}{\varvec{\beta}}+{\varvec{Z}}{\varvec{U}}{\varvec{\gamma }}+{\varvec{Z}}{\varvec{b}})\right\} ^{\mathrm{T}}{\varvec{X}}=0,$$

5 $$\left\{ {\varvec{Y}}-\exp ({\varvec{X}}{\varvec{\beta}}+{\varvec{Z}}{\varvec{U}}{\varvec{\gamma }}+{\varvec{Z}}{\varvec{b}})\right\} ^{\mathrm{T}}{\varvec{Z}}{\varvec{U}}=0,$$

and

6 $$\left\{ {\varvec{Y}}-\exp ({\varvec{X}}{\varvec{\beta}}+{\varvec{Z}}{\varvec{U}}{\varvec{\gamma }}+{\varvec{Z}}{\varvec{b}})\right\} ^{\mathrm{T}}{\varvec{Z}}= {\varvec{b}}^{\mathrm{T}}{\varvec{D}}^{-}.$$

Iterative re-weighted least squares (IRLS) can be applied to solve the above equations for ${\varvec{\beta}}$, ${\varvec{\gamma }}$ and ${\varvec{b}}$. However, high computational costs and memory space constraints often make it difficult to apply these iterative procedures to data sets with a very large number of cases. An alternative computational strategy is the use of the Gauss–Seidel algorithm. In this method, at each iteration, one of the parameters is estimated while keeping other parameters fixed at current values. The advantage of such an approach is that substantial simplifications can be obtained at each step. Using this approach, we first initialize ${\varvec{\beta}}$ and then obtain updated estimates for ${\varvec{\gamma }}$ and ${\varvec{b}}$ in the following two step procedure:

#### *Step 0*

 Set the coefficients corresponding to area level covariates, ${\varvec{\gamma }}$ and random effects, ${\varvec{b}}$ to ${\varvec{0}}$ in Eq. (4). Then we have

$$\begin{aligned} \left\{ {\varvec{Y}}-\exp ({\varvec{X}}{\widehat{\varvec{\beta}}})\right\} ^{\mathrm{T}}{\varvec{X}}=0. \end{aligned}$$

This equation is the estimating equation for a Poisson generalized linear model [14] and thus can be fitted using the existing glm function in the ${\varvec{R}}$ statistical computing environment [15]. This gives initial estimates of the regression coefficient ${\varvec{\beta}}$ associated with individual level covariates.

#### *Step 1*

 Substitute the current estimated individual level coefficients, ${\widehat{\varvec{\beta}}}$ in Eqs. (5) and (6) and with some simple algebra, we have

$$\left\{ {\varvec{Y}}_c-\exp (\text {O}_1+{\varvec{U}}{\varvec{\gamma }}+{\varvec{b}})\right\} ^{\mathrm{T}}{\varvec{U}}=0$$

and,

$$\left\{ {\varvec{Y}}_c-\exp (\text {O}_1+{\varvec{U}}{\varvec{\gamma }}+{\varvec{b}})\right\} ^{\mathrm{T}}={\varvec{b}}^{\mathrm{T}}{\varvec{D}}^{-},$$

where ${\varvec{Y}}_c^{\mathrm{T}}= {\varvec{Y}}^{\mathrm{T}}{\varvec{Z}}$ is a vector of aggregated disease counts of length *M* at the group level and $\text {O}_1=\log \{{\varvec{Z}}^{\mathrm{T}}\exp ({\varvec{X}}{\widehat{{\varvec{\beta}}}})\}$ is a vector of offset with length *M*.

The above two equations are well known PQL estimating equations for the Poisson mixed model [13]. Since, the outcome ${\varvec{Y}}_c$, offset $\text {O}_1$, covariate ${\varvec{U}}$ and random effects ${\varvec{b}}$ are all measured at the group level, estimates of parameters for the group level coefficient ${\widehat{{\varvec{\gamma }}}}$ and random effects ${\varvec{b}}$ can be estimated using the PQL method [7, 13] with only group level data. The detailed procedure is described in Appendix 1.

#### *Step 2*

 Now substitute the estimated area-specific regression coefficient, ${\widehat{{\varvec{\gamma }}}}$ and random effect parameter, ${\widehat{\varvec{b}}}$ estimated at step 1 in (4). Then we have

$$\left\{ {\varvec{Y}}-\exp ({\varvec{X}}{\varvec{\beta}}+\text {O}_2)\right\} ^{\mathrm{T}}{\varvec{X}}=0,$$

where $\text {O}_2={\varvec{Z}}({\varvec{U}}{\widehat{\varvec{\gamma}}}+{\widehat{\varvec{b}}})$ is an offset vector of length *n*. Under the above specification, the individual level coefficients estimate ${\widehat{{\varvec{\beta}}}}$ can then be updated using ordinary Poisson regression with individual level data.

Steps 1 and 2 are then repeated until the algorithm converges. Estimates obtained by this iterative procedure will be the same, aside from rounding error as the solution obtained by a standard IRLS algorithm.

#### Estimation of standard error

The approximate standard error estimates for ${\widehat{{\varvec{\gamma }}}}$ and ${\widehat{\varvec{\beta}}}$ in steps 1 and 2 assume fixed ${{\varvec{\beta}}}$ and fixed ${{\varvec{\gamma }}}$, respectively. Therefore, we re-calculated the standard error of these regression coefficients by adjusting the variability of the estimated ${\widehat{\varvec{\beta}}}$ and ${\widehat{\varvec{\gamma}}}$. This can be done via the IRLS estimation of score equations (4–6). The IRLS estimation requires us to define a working dependent variable and a weight matrix that are updated at each iteration and solved via Fisher scoring [13].

Let the GLM adjusted dependent variable, ${\varvec{Y}}_{pseudo}$ be

7 $${\varvec{Y}}_{pseudo}= {} {\varvec{X}}{\varvec{\beta}}+{\varvec{Z}}{\varvec{U}}{\varvec{\gamma }}+{\varvec{Z}}{\varvec{b}}+{\varvec{W}}^{-1}({\varvec{Y}}-{\varvec{\mu }})$$

where ${\varvec{W}}$ is a $n\times n$ diagonal matrix with diagonal elements ${\varvec{\mu }}$. Harville [16] and Robinson [17] showed that the Fisher scoring corresponding to the score equations (4–6) and GLM dependent variable as in (7), is identical to the normal equation of the best linear unbiased predictors (BLUPs) of ${\varvec{\beta}}$, ${\varvec{\gamma }}$ and ${\varvec{\theta }}$ corresponding to the following linear mixed model

8 $$\begin{aligned} {\varvec{Y}}_{pseudo} = {\varvec{X}}{\varvec{\beta}}+{\varvec{Z}}{\varvec{U}}{\varvec{\gamma }}+{\varvec{Z}}{\varvec{b}}+\varvec{\epsilon }_{pseudo}, \end{aligned}$$

where the pseudo-error $\varvec{\epsilon }_{pseudo}\sim N(0,{\varvec{W}}^{-1})$. Following [17], the estimated regression coefficients for the fixed effects, $({\varvec{\beta}}, {\varvec{\gamma }})$ and BLUP estimate for the random effect ${\varvec{b}}$ can be obtained as

9 $$\begin{aligned} \left({\widehat{\varvec{\beta}}}, {\widehat{{\varvec{\gamma }}}}\right)&= {} \left( {\varvec{C}}^{\mathrm{T}}{\varvec{V}}^{-1} {\varvec{C}}\right) ^{-1} \left( {\varvec{C}}^{\mathrm{T}}{\varvec{V}}^{-1} {\varvec{Y}}_{pseudo}\right) \nonumber \\ {\widehat{\varvec{b}}}&= {} {\varvec{D}}{\varvec{Z}}^{\mathrm{T}}{\varvec{V}}^{-1}\left\{ {\varvec{Y}}-{\varvec{X}}{\widehat{\varvec{\beta}}}-{\varvec{Z}}{\varvec{U}}{\widehat{\varvec{\gamma}}}\right\} \end{aligned}$$

where ${\varvec{C}}=[X|ZU]$ and ${\varvec{V}}={\varvec{Z}}{\varvec{D}}{\varvec{Z}}^{\mathrm{T}}+{\varvec{W}}^{-1}$, the variance of pseudo-response ${\varvec{Y}}_{pseudo}$. Thus, the variance–covariance matrix for the fixed effect $({\widehat{\varvec{\beta}}}, {\widehat{\varvec{\gamma}}})$ can be estimated by

10 $${\varvec{Q}}=\left( {\varvec{C}}^{\mathrm{T}}{\varvec{V}}^{-1} {\varvec{C}}\right) ^{-1}.$$

Note that Eq. (9) suggests that estimates of the regression coefficients and variance components can be obtained using the Leroux et al. [7] model with appropriate specification of the design matrix (${\varvec{Z}}$) associated with spatial random effect (1). Indeed, a back-fitting approach such as indiCAR will be effective in situations where memory constraints may prohibit fitting a single model consisting of all individual and group level covariates. A useful feature of our indiCAR method is that we can calculate the above standard error in a distributed computing framework. This is because ${\varvec{V}}^{-1}$ can be expressed as ${\varvec{W}}- {\varvec{W}}{\varvec{Z}}{\varvec{D}}(I + {\varvec{Z}}^{\mathrm{T}}{\varvec{W}}{\varvec{Z}}{\varvec{D}})^{-1}{\varvec{Z}}^{\mathrm{T}}{\varvec{W}}$ [18]. Therefore, the above variance–covariance matrix can be written as

$$\begin{aligned} {\varvec{Q}}=\left( \begin{array}{cc} a_{11} &{}\quad a_{12}\\ a_{21} &{}\quad a_{22} \end{array} \right) ^{-1}, \end{aligned}$$

where $a_{11}={\varvec{X}}^{\mathrm{T}}{\varvec{W}}{\varvec{X}}- {\varvec{X}}^{\mathrm{T}}{\varvec{W}}{\varvec{Z}}{\varvec{D}}(\varvec{I}+{\varvec{Z}}^{\mathrm{T}}{\varvec{W}}{\varvec{Z}}{\varvec{D}})^{-1}\times {\varvec{Z}}^{\mathrm{T}}{\varvec{W}}{\varvec{X}}$, $a_{12}={\varvec{X}}^{\mathrm{T}}{\varvec{W}}{\varvec{Z}}{\varvec{U}}- {\varvec{X}}^{\mathrm{T}}{\varvec{W}}{\varvec{Z}}{\varvec{D}}(\varvec{I}+{\varvec{Z}}^{\mathrm{T}}{\varvec{W}}{\varvec{Z}}{\varvec{D}})^{-1}\times {\varvec{Z}}^{\mathrm{T}}{\varvec{W}}{\varvec{Z}}{\varvec{U}}$, $a_{21}= a_{12}^{\mathrm{T}}$, $a_{22}={\varvec{U}}^{\mathrm{T}}{\varvec{Z}}^{\mathrm{T}}{\varvec{W}}{\varvec{Z}}{\varvec{U}}- {\varvec{U}}^{\mathrm{T}}{\varvec{Z}}^{\mathrm{T}}{\varvec{W}}{\varvec{Z}}{\varvec{D}}\times (\varvec{I}+{\varvec{Z}}^{\mathrm{T}}{\varvec{W}}{\varvec{Z}}{\varvec{D}})^{-1}{\varvec{Z}}^{\mathrm{T}}{\varvec{W}}{\varvec{Z}}{\varvec{U}}$. Among the various components of the above variance-covariance matrix, ${\varvec{X}}^{\mathrm{T}}{\varvec{W}}{\varvec{X}}$ and ${\varvec{X}}^{\mathrm{T}}{\varvec{W}}{\varvec{Z}}$ are the only terms involving individual level data, and the rest of the terms involve a lower dimension corresponding to the group level data. These components are therefore straightforward to calculate. Hence, upon convergence, calculation of the variance–covariance matrix is also carried out in a distributed computing framework for individual and group-level data separately.

The covariance matrix for ${\widehat{\varvec{b}}}$ was obtained from the Fisher information matrix from step 2 in the usual way, assuming that parameters for the individual and area specific covariates are fixed. Of course there is additional variability due to the fact that the individual and area specific covariates parameters are estimated. However, following Breslow and Clayton [13] we ignore this additional variability when making inference about the parameters which characterise the random effect distribution, ${\widehat{{\varvec{\theta }}}}$. The detailed procedure is given in Appendix 1.

In the next section we describe a simulation study to evaluate the performance of our method.

## Simulation studies

To evaluate our proposed method we design a simulation study involving 400 regions in a $20 \times 20$ square lattice grid with varying sample sizes. Specifically, we consider cases with (i) 10–1000 and (2) 10–50 subjects in each area. We declare two regions to be neighbours if they share a common border. The random effects are generated following a multivariate normal distribution with mean 0 and covariance matrix ${\varvec{D}}=[\sigma ^{-2}\left\{ (1-{\varvec{\lambda }})\varvec{I}+{\varvec{\lambda }}{\varvec{R}}\right\} ]^{-1}$. The value of $\sigma$ is set to 0.4 and five different values of spatial dependence parameters, ${\varvec{\lambda }}= \{0, 0.25,0.50, 0.75, 0.99\}$ are considered in order to represent different strengths of spatial correlation. We then generate three individual level covariates (one binary, one categorical and one continuous) and one group level covariate. The binary covariate represents the distribution of sex in the area and is generated following a Bernoulli random variable with probability ranging from 0.45 to 0.55 across groups. The categorical variable with six categories is generated to represent the age distribution of the neutropenia admissions data with prespecified probabilities (similar to the neutropenia admissions data). The continuous individual level variable is generated as Uniform (0.2, 1). The group level covariate is generated from a standard normal distribution. The outcome variable is then generated using model (1). The full list of the parameters used to generate data is given in Table 1. The binary and the categorical individual level variables help us to compare our simulation results for the indiCAR with the age-sex adjusted Leroux et al. [7] approach.

**Table 1:** Simulation results for estimated regression coefficients following indiCAR and Leroux et al. [7] where each area consists of a random number of subjects between 10 and 1000

True value	indiCAR											Leroux et al. [7] approach		
	$\beta _0$ −0.20	$\beta _1$ −2.50	$\beta _2$ 0.70	$\beta _{32}$ −2.00	$\beta _{33}$ −1.50	$\beta _{34}$ 0.20	$\beta _{35}$ 0.50	$\beta _{36}$ 0.80	$\gamma$ 0.20	$\sigma$ 0.40	$\lambda$	$\gamma$ 0.20	$\sigma$ 0.40	$\lambda$
$\lambda$	Estimated coefficient													
0.00	−0.178	−2.500	0.700	−1.997	−1.500	0.201	0.501	0.800	0.199	0.396	0.019	0.198	0.442	0.069
0.25	−0.174	−2.499	0.699	−1.997	−1.498	0.200	0.501	0.801	0.198	0.395	0.251	0.198	0.421	0.304
0.50	−0.162	−2.500	0.700	−2.001	−1.501	0.200	0.500	0.801	0.198	0.396	0.503	0.198	0.413	0.523
0.75	−0.152	−2.501	0.701	−2.005	−1.499	0.201	0.500	0.800	0.198	0.394	0.736	0.198	0.407	0.722
0.99	−0.144	−2.499	0.700	−2.000	−1.500	0.200	0.499	0.799	0.199	0.396	0.958	0.199	0.412	0.950
	Empirical standard error													
0.00	0.033	0.016	0.012	0.059	0.039	0.025	0.026	0.026	0.021	0.030	0.028	0.022	0.035	0.056
0.25	0.037	0.016	0.011	0.060	0.039	0.027	0.026	0.027	0.017	0.033	0.101	0.017	0.035	0.106
0.50	0.042	0.016	0.012	0.058	0.040	0.027	0.027	0.026	0.016	0.029	0.130	0.016	0.028	0.120
0.75	0.054	0.016	0.012	0.061	0.039	0.028	0.027	0.028	0.013	0.025	0.114	0.014	0.025	0.106
0.99	0.209	0.017	0.012	0.064	0.038	0.027	0.027	0.027	0.012	0.020	0.037	0.013	0.020	0.043
	Average of the simulated standard error													
0.00	0.034	0.016	0.012	0.061	0.039	0.027	0.027	0.027	0.021	0.016	0.026	0.022	0.018	0.031
0.25	0.036	0.016	0.012	0.061	0.039	0.028	0.027	0.027	0.017	0.017	0.053	0.018	0.018	0.058
0.50	0.041	0.016	0.012	0.062	0.039	0.028	0.027	0.027	0.015	0.018	0.079	0.015	0.018	0.080
0.75	0.028	0.016	0.012	0.062	0.039	0.028	0.027	0.027	0.014	0.019	0.086	0.014	0.019	0.086
0.99	0.130	0.016	0.012	0.062	0.040	0.028	0.027	0.028	0.013	0.019	0.034	0.013	0.020	0.038

## Results and discussion

In this section we discuss our results obtained from the simulation study and present an application to the neutropenia admissions data. We compare the results obtained by indiCAR to those from the existing Leroux et al. [7] method. When applying indiCAR to the simulated data, we adjust for all individual and areal covariates. However, in the existing Leroux et al. [7] method we were only able to incorporate the binary and categorical variable by calculating offsets based on direct standardization of these covariates.

### Simulation results

Table 1 displays the average estimated regression coefficients along with their estimated standard errors for the indiCAR and Leroux et al. [7] methods based on 1000 simulation runs based on simulation scenario (1). We estimated two different standard errors of estimated regression coefficients: namely, (1) empirical standard errors i.e., taking the standard deviation of the 1000 simulated regression coefficient estimates, (2) average of model based standard errors. The first column of Table 1 specifies the spatial dependence parameter used in that particular simulation. The next eight columns list the estimated regression coefficients for the individual level covariates using the indiCAR method. The 10th, 11th and 12th columns list the estimated group level regression coefficients, the estimated overdispersion parameters and estimated spatial dependence parameters for the spatial random effect using the indiCAR method. The last three columns list the estimated regression coefficients for the group specific covariate and estimated overdispersion and spatial dependence parameters using the Leroux et al. [7] method. The Leroux et al. [7] method adjusts only for the binary and categorical variables.

As expected, the indiCAR method provides reliable estimates of the individual level and region specific regression parameters and the parameters in the spatial random effect. Although the Leroux et al. [7] method provides similar reliable estimates of the true region-specific regression parameters, the random effect parameters are slightly biased.

To evaluate the performance of the proposed method under small sample settings, we also conducted simulations with only 10–50 subjects per region as outlined in simulation scenario (2).These results are given in Table 2. As indicated in the table, the proposed method performs very well in this setting, providing reliable estimates of all the parameters. In contrast, the Leroux et al. [7] method provides slightly less efficient estimates of the spatial dependence parameters.

**Table 2:** Simulation results for estimated regression coefficients following indiCAR and Leroux et al. [7] where each area consists of a random number of subjects between 10 and 50

True value	indiCAR											Leroux et al. [7] method		
	$\beta _0$ −0.20	$\beta _1$ −2.50	$\beta _2$ 0.70	$\beta _{32}$ −2.00	$\beta _{33}$ −1.50	$\beta _{34}$ 0.20	$\beta _{35}$ 0.50	$\beta _{36}$ 0.80	$\gamma$ 0.20	$\sigma$ 0.40	$\lambda$	$\gamma$ 0.20	$\sigma$ 0.40	$\lambda$
$\lambda$	Estimated coefficient													
0.00	−0.161	−2.495	0.697	−2.000	−1.481	0.210	0.509	0.811	0.197	0.380	0.043	0.189	0.444	0.059
0.25	−0.175	−2.501	0.699	−2.020	−1.500	0.205	0.505	0.804	0.197	0.382	0.247	0.193	0.426	0.225
0.50	−0.179	−2.500	0.701	−2.011	−1.509	0.199	0.501	0.802	0.199	0.380	0.462	0.195	0.430	0.396
0.75	−0.177	−2.504	0.700	−2.038	−1.504	0.207	0.507	0.806	0.200	0.380	0.656	0.195	0.468	0.496
0.99	−0.156	−2.498	0.700	−2.039	−1.505	0.204	0.505	0.802	0.199	0.402	0.929	0.197	0.510	0.856
	Empirical standard error													
0.00	0.117	0.148	0.056	0.301	0.184	0.115	0.114	0.123	0.032	0.064	0.070	0.033	0.065	0.078
0.25	0.086	0.050	0.038	0.200	0.120	0.086	0.081	0.083	0.026	0.047	0.154	0.027	0.053	0.147
0.50	0.085	0.049	0.039	0.183	0.128	0.083	0.081	0.086	0.025	0.042	0.185	0.026	0.048	0.179
0.75	0.125	0.071	0.051	0.260	0.163	0.118	0.110	0.115	0.027	0.051	0.200	0.029	0.056	0.217
0.99	0.225	0.064	0.051	0.259	0.163	0.118	0.116	0.124	0.029	0.052	0.094	0.031	0.058	0.147
	Average of the simulated standard error													
0.00	0.116	0.066	0.049	0.254	0.160	0.114	0.112	0.113	0.031	0.036	0.075	0.032	0.035	0.062
0.25	0.092	0.051	0.038	0.197	0.125	0.088	0.086	0.087	0.025	0.033	0.114	0.027	0.032	0.096
0.50	0.093	0.052	0.038	0.197	0.125	0.088	0.086	0.087	0.024	0.036	0.163	0.025	0.035	0.134
0.75	0.122	0.067	0.049	0.261	0.163	0.115	0.113	0.114	0.028	0.051	0.191	0.029	0.047	0.163
0.99	0.164	0.067	0.049	0.260	0.163	0.115	0.112	0.114	0.027	0.051	0.059	0.029	0.052	0.088

Following reviewer suggestions, we also compared the indiCAR method with three other methods; a group specific random intercept model (1) using the *lme4* [19] and (2) using the *hglm* [20] packages in *R* and (3) a CAR model implemented using the *hlmer* function in the *hglmR* package. The three methods were compared in terms of both approximate conditional AIC [21] and computation time. The results are given in Tables 3 and 4. In Table 3, the data were generated with $\lambda =0$, which means that a random intercept only model is appropriate. In Table 4, the data were generated with $\lambda =0.75$, which means that a CAR component is necessary for an accurate model fit. Note that the conditional AIC values are approximate as these are calculated ignoring the constant term in the log-likelihood. The *hlmer* approach is faster when block effects are represented by a random intercept, but is slower for a conditional autoregressive random effect specification. The fitting of *hlmer* with such a random effect specification is not even feasible for large sample sizes on our standard desktop computer due to large memory requirements. In addition, we note that another R package: *sdep* has similar feasibility issues when fitting a conditional autoregressive random effect model for large datasets [22]. Our proposed indiCAR method in general provides lower conditional AIC compared to other models considered here and is faster than the *hlmer* approach when using a CAR random effect specification, as we do in our application.

**Table 3:** Comparison of estimated time and conditional AIC between indiCAR and other methods when data are generated without spatial random effect, ${\varvec{\lambda }}=0$

Sample per group	Total sample	Time to convergence (s)				Conditional AIC			
		indiCAR	glmer with random intercept	hlmer with random intercept	hlmer with CAR	indiCAR	glmer with random intercept	hlmer with random intercept	hlmer with CAR
Data generated in 100 groups									
1:50	2373	0.73	1.98	0.43	2.36	1419.26	1492.26	1445.65	1445.87
1:100	5056	2.09	5.26	0.55	2.93	3170.9	3225.28	3194.03	3193.98
1:500	26,473	10.34	23.63	1.65	12.96	15,996.05	15,968.02	15,955.41	15,955.40
1:1000	48,778	37.25	53.25	3.01	29.68	31,063.34	31,011.67	31,001.58	31,001.72
Data generated in 400 groups									
1:50	10,192	51.39	9.44	2.64	97.45	6027.28	6242.24	6097.72	6097.84
1:100	19,843	73.31	33.13	11.39	244.02	12,017.28	12,185.15	12,037.20	12037.27
1:500	98,870	140.74	71.96	38.91	Not feasible	59,061.39	58,929.01	58,879.30	Not feasible
1:1000	205,952	207.84	214.51	149.96	Not feasible	121,733.50	121,533.80	121,510.20	Not feasible

**Table 4:** Comparison of estimated time and conditional AIC between indiCAR and other methods when data are generated with spatial random effect parameter, ${\varvec{\lambda }}=0.75$

Sample per group	Total sample	Time to convergence (s)				Conditional AIC			
		indiCAR	glmer with random intercept	hlmer with random intercept	hlmer with CAR	indiCAR	glmer with random intercept	hlmer with random intercept	hlmer with CAR
Data generated in 100 groups									
1:50	2517	0.48	2.34	0.39	2.10	1748.78	1883.67	1881.69	1881.81
1:100	4688	3.30	3.40	0.38	6.75	2821.61	2899.41	2897.73	2897.83
1:500	26,519	4.23	23.71	1.92	15.52	15,865.50	15,943.65	15,943.58	15,943.55
1:1000	52,911	188.19	61.84	3.62	Not feasible	32,632.45	32,669.39	32,669.18	Not feasible
Data generated in 400 groups									
1:50	10,118	51.55	14.81	2.65	138.33	5935.14	6323.31	6309.56	6309.44
1:100	20,652	36.74	25.53	4.20	434.66	12,476.61	12,893.85	12,889.04	12,889.13
1:500	103,267	85.75	73.45	22.31	Not feasible	60,233.22	60,533.49	60,533.24	Not feasible
1:1000	205,739	113.65	236.95	46.23	Not feasible	120,212.20	120,423.70	120,423.00	Not feasible

### Application to the neutropenia data

We applied our methodology to the data on neutropenia admissions. One of the key objectives of this analysis is to assess the geographical variation of neutropenia admission rates and its association with area level measures of socioeconomic status. Data also includes patient age, gender, year of diagnosis, ARIA, cancer type at diagnosis, number of major comorbidities and geographic location reported via postcode of residence.

Table 5 shows the descriptive statistics for cancer patients treated between years 2001 and 2009 in New South Wales, Australia. The proportion of neutropenia admissions decreases gradually with increasing age (9.2 % for 20–30 years of age to 1.7 % for 80+ years of age). Overall, the rates are similar (≈5 %) across the years 2001–2008 but are considerably lower (3.0 %) in the year 2009. This is likely due to the fact that the data are date limited for those patients diagnosed with cancer and treated with chemotherapy in 2009. Cancer treatment often has a long duration, and subsequent neutropenia admissions may have happened beyond the study period. The proportion of neutropenia is highest (4.9 %) in the major cities followed by inner regional Australia (3.9 %). Among the various types of cancer, the highest proportion of neutropenia admissions are observed for haematological malignant cancer patients (25.0 %) followed by lung (6.2 %) and breast cancer (5.3 %). The proportion of neutropenia admissions are very similar across various SEIFA index categories.

**Table 5:** Descriptive analsis of neutropenia data

Variables	Neutropenia n (%)	Total
Age group (years)		
20–30	408 (9.2)	4418
30–39	851 (7.7)	10,988
40–49	1649 (6.2)	26,395
50–59	2942 (5.6)	52,281
60–69	3465 (4.8)	71,446
70–79	2577 (3.7)	69,236
80+	769 (1.7)	44,859
Sex		
Female	6363 (5.0)	127,519
Male	6298 (4.1)	152,104
Year of diagnosis		
2001	1343 (4.9)	27,356
2002	1411 (5.0)	28,451
2003	1503 (5.1)	29,560
2004	1478 (4.8)	30,970
2005	1596 (5.1)	31,533
2006	1452 (4.6)	31,865
2007	1453 (4.5)	32,603
2008	1405 (4.2)	33,343
2009	1020 (3.0)	33,942
ARIA		
Major cities	9199 (4.9)	189,322
Inner regional Australia	2638 (3.9)	67,086
Outer regional Australia	774 (3.6)	21,664
Remote or very remote Australia	50 (3.2)	1551
Cancer type		
Breast cancer	2059 (5.3)	38,620
Lung cancer	1401 (6.2)	22,744
Colon and rectum cancer	1011 (3.0)	34,018
Haematological malignancy	5134 (25.0)	20,518
Other cancer	3056 (1.9)	163,723
No. of major comorbidities		
0	6072 (3.7)	163,645
1	2228 (4.9)	45,817
2	2315 (6.7)	34,670
3	976 (5.7)	17,264
4+	1,070 (5.9)	18,227
SEIFA		
Most disadvantaged	1388 (4.6)	30,302
2	1750 (4.1)	42,558
3	3546 (4.5)	78,006
4	2800 (4.6)	60,880
Least disadvantaged	3177 (4.7)	67,877

Table 6 reports the multivariable analysis of neutropenia admissions data using both the indiCAR approach and the Leroux et al. [7] method based on age-sex adjustments. We calculate age-sex adjusted standardized incidence ratios (SIR) by dividing the observed number of neutropenia admissions by the age-sex adjusted expected number of neutropenia admissions [23]. Our results reveal significantly lower rates of neutropenia for patients with higher age, male gender, residence in an outer regional or remote area and higher socioeconomic status. The estimated overdispersion $(\sigma )$ and spatial dependence parameters $(\lambda )$ with indiCAR are 0.204 and 0.992, respectively compared to 0.210 and 0.989 for the Leroux et al. [7] method. This means that both models identified a very strong spatial correlation in the neutropenia risk.

**Table 6:** Comparison of individual covariate adjusted conditional autoregressive model (indiCAR) with the Leroux et al. [7] method based on age-sex adjustments

Regression coefficients	indiCAR		Leroux et al.	
	Estimates	SE	Estimates	SE
Intercept	−2.781	0.110	–	–
Age group (years)				
20–30	0.124	0.056	–	–
30–39	0.208	0.042	–	–
40–49	Ref.			
50–59	−0.119	0.031	–	–
60–69	−0.287	0.031	–	–
70–79	−0.712	0.033	–	–
80+	−1.586	0.045	–	–
Sex				
Female	Ref.			
Male	−0.082	0.020	–	–
Year of diagnosis				
2001	Ref.			
2002	0.018	0.038	–	–
2003	0.083	0.038	–	–
2004	0.021	0.038	–	–
2005	0.096	0.037	–	–
2006	0.036	0.038	–	–
2007	0.026	0.038	–	–
2008	−0.001	0.038	–	–
2009	−0.315	0.042	–	–
ARIA				
Major cities	Ref.			
Inner regional Australia	−0.023	0.047	–	–
Outer regional Australia	−0.147	0.068	–	–
Remote/very remote Australia	−0.231	0.163	–	–
Cancer type				
Breast cancer	Ref.	–	–	–
Lung cancer	0.253	0.038	–	–
Colon and rectum cancer	−0.434	0.040	–	–
Haematological malignancy	1.572	0.029	–	–
Other cancer	−0.942	0.031	–	–
No. of major comorbidities				
0	Ref.	–	–	
1	0.413	0.026	–	–
2	0.670	0.026	–	–
3	0.609	0.036	–	–
4+	0.605	0.035	–	–
SEIFA				
Most disadvantaged	Ref.			
2	−0.083	0.044	−0.075	0.042
3	−0.071	0.041	−0.068	0.038
4	−0.125	0.047	−0.121	0.044
Least disadvantaged	−0.131	0.056	−0.129	0.052
Variance parameter				
*σ*	0.204	0.022	0.210	0.022
*λ*	0.992	0.012	0.989	0.015

Although advanced age has been identified as a significant predictor for neutropenia admissions in previous studies [24], we observed a lower risk of neutropenia admissions associated with increasing age. This might be due to the fact that the current guidelines for prophylactic administration of colony stimulating factor (CSF) already account for age [25]. CSF is an effective treatment strategy to reduce neutropenia.

The relationship between average neutropenia rates and ARIA and SEIFA are in the opposite direction, which is counter intuitive as remote areas in NSW are mostly associated with disadvantaged SEIFA categories. However, the observed contrast in estimated regression coefficients might be due to the differences in the health care practices. Patients in the remote areas are likely to be geographically distant to the treating medical oncologist and hence managed by their primary care physicians. Consequently, these patients may be treated with lower doses of chemotherapy [26]. On the contrary, patients in the major cities might get intensive and aggressive chemotherapy, and are better managed due to availability of resources. Previous studies also indicate that remoteness has a great effect on the quality of cancer treatment [27] and that it affects treatment choices made by both patients and clinicians [28].

**Fig. 1:**
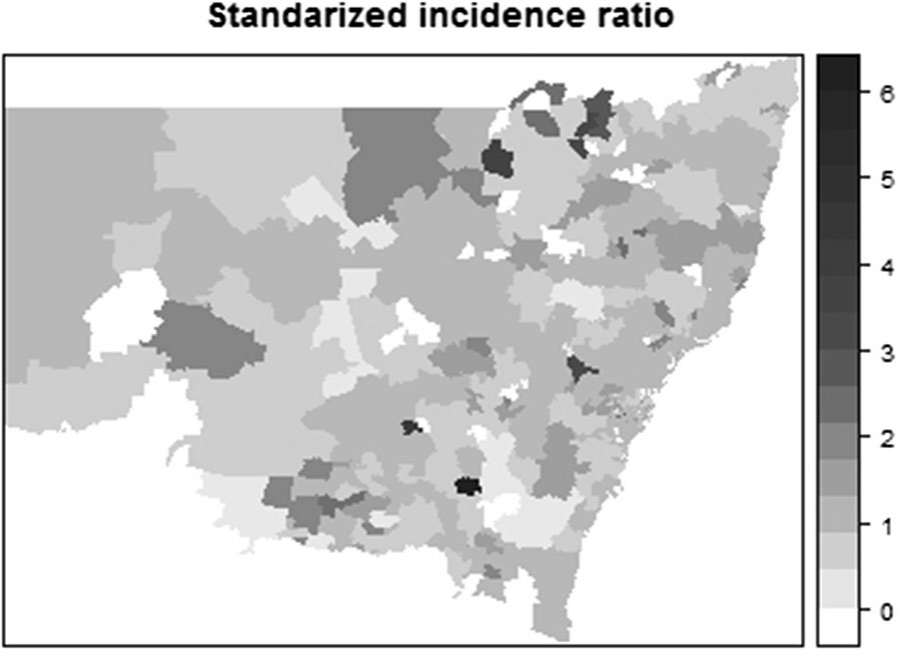
SIR of neutropenia admissions in NSW region following indiCAR

**Fig. 2:**
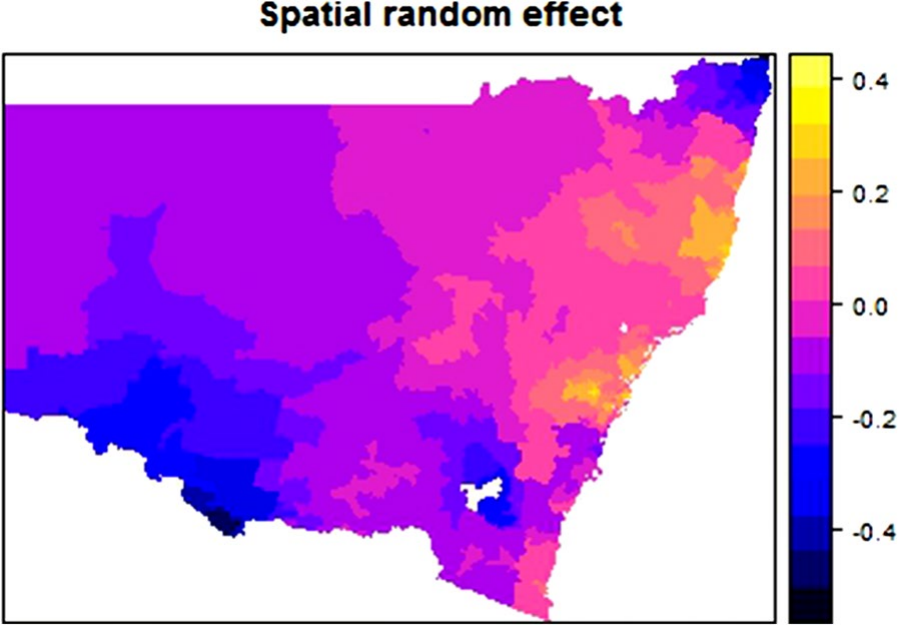
Estimated spatial random effect across NSW using proposed indiCAR method

Figure 1 shows the SIR of neutropenia admissions in NSW. Six postal areas in NSW had an estimated SIR >3 as shown in the map. Figure 2 shows a that neutropenia rates across NSW exhibit a very high spatial dependence. The white region in the map of NSW is the Australian Capital Territory (ACT), which is a distinct territory not included in our dataset. Two other Australian states, Queensland (QLD) and Victoria (VIC) are located to the North-East and South-West of NSW, respectively. The strong spatial correlation after adjusting for individual and group specific covariates indicates that geographical variation of neutropenia might be due to differences in health care practices or access to care across NSW. Further investigation at the hospital level would be needed in order to provide a comprehensive explanation of these findings. In some cases, a lower spatial random effect might be the result of low numbers of cancer patients being recruited in our study due to a border effect (i.e., getting admitted for neutropenia in other states: ACT, Victoria or Queensland) or due to areas being dominated by private cancer facilities.

Variation across clinical practices of neutropenia have been identified in Australia in a previous survey [29]. The authors showed that the treatment approach for management of neutropenia varies across oncologists, hematologists and clinicians as well as different sectors of cancer care. Therefore, it might be interesting to explore whether the observed variation is due to variation across different hospitals (for example, metropolitan vs. non-metropolitan hospitals) in NSW or across various healthcare providers. However, relevant data for such analysis are not collected in the registry and further exploration is beyond the scope of our present paper.

Our study was based on data linked from a state-based cancer registry and administrative data from the APDC. An advantage of such linked data is that it provides us with a large, population based sample. Registry based analysis is more comprehensive than that based on single centre studies, and provides more complete information than may be obtained from clinical trials where patient selection and loss to follow-up may impact validity and generalizability of study findings. However, it is important to keep in mind that the resulting data quality may be inferior to that obtained from prospective studies.

We should note that in some cases, separate admissions for the same individual may be correlated, and thus the Poisson assumptions for the number of admissions may not be appropriate. In such cases, one could fit a subject specific random effect model at the individual level data rather than a generalized linear model [30]. In our application, we do not have such issues, because neutropenia is a very rare event and we do not have any cases with recurrent neutropenia admissions. Therefore, it is suitable to use a Poisson approximation to the Binomial distribution for our dataset.

In our simulations, the estimation of the intercept $\beta _0$ is biased. This is consistent with the observation of Hodges and Reich [31] that an intercept is poorly identified in the model with the presence of spatial random effect. The authors further argued that adding spatially correlated errors can attenuate the fixed effect estimation. However, they only considered one observation per areal unit rather cases with replicated data such as that in our application. There may be other explanations for attenuations, for example, Huque et al. [32] argued that such attenuation is likely due to covariate measurement error.

Despite various limitations, indiCAR is an useful addition to the existing methodology to explore clinical variation across geographical locations. One of the major advantages of our proposed method is the ability to analyze age as a continuous variable rather than grouping them using an arbitrary cut-off. The results of such an analysis are given in Appendix 2, though they are very similar to those using age groups. However, in many applications age grouping might induce residual confounding and result in spurious relationships between age and the outcome variable [33]. In our simulation study, we evaluate our proposed method for a continuous area level covariate; however, interpretation of the SEIFA index is difficult as a continuous variable. Therefore, to ease our interpretation we considered SEIFA as a categorical variable. We also conducted an analysis of neutropenia admissions data using continuous SEIFA index. The results are quite similar and indicate a significant negative relationship between high SEIFA score and neutropenia admissions (result not shown in table).

## Conclusions

In this paper we propose a novel method for incorporating individual level covariate information in disease mapping studies. As indicated in our simulation studies, our proposed method yields reliable estimates of individual and area level covariate effects. Our proposed method also has potential for Big Data implementations due the natural applicability of indiCAR in a distributed computing framework. This could speed up the process and reduce large computational costs. Furthermore, indiCAR also provides a framework for fitting correlated Big Data using recently developed statistical methodology for uncorrelated Big Data [34, 35]. Cancer registries routinely collect individual level cancer information and thus could benefit by using our proposed method to incorporate individual level information in the analysis and mapping of disease rates.

## Appendix 1: Implementation of PQL in step 1

The PQL estimation procedure is a iterative approach where at each step one must define a working dependent variable and a weight matrix which are then updated at each iteration and solved via Fisher scoring [7, 13]. The detailed procedure has been illustrated elsewhere [7, 13].

The GLM adjusted dependent variable (${\varvec{Y}}_{c-pseudo}$) at group level is calculated as

11 $${\varvec{Y}}_{c-pseudo}={\hat{\eta}}_c+({\varvec{Y}}_c-\hat{\mu }_c)\frac{d {\hat{\eta} _c}}{d{\hat{\mu} _c}},$$

where $\eta _c=g(\mu _c)=\text {O}_1+{\varvec{U}}{\varvec{\gamma }}+{\varvec{b}}$ and $\text {O}_1=\log \{{\varvec{Z}}^{\mathrm{T}}\exp ({\varvec{X}}{\widehat{\varvec{\beta}}})\}$ is an offset vector with dimension $M$. The Poisson link $(g(\mu _c)=\log \mu _c)$ and variance function $V(\mu _c)=\mu _c$ are used. The covariance matrix of ${\varvec{Y}}_{c-pseudo}$ is then approximated by

12 $${\widehat{V}}_c={\widehat{W_c}}^{-1}+{\widehat{D}},$$

where ${\hat{D}}$ is the covariance matrix of the random effects, ${\varvec{b}}$, evaluated at the current estimate for the variance parameters and ${\hat{W}}_c$ is the $M\times M$ diagonal matrix with diagonal elements ${\hat{\mu }}_c$. Updated estimates of the fixed effect vector ${\varvec{\gamma }}$ and random effect vector ${\varvec{b}}$ are then can be obtained from the solution of the following mixed model equations:

13 $${\widehat{{\varvec{\gamma }}}}=({\varvec{U}}^{\mathrm{T}}{\widehat{V_c}}^{-1}{\varvec{U}})^{-1}{\varvec{U}}{\widehat{V_c}}^{-1}\left( {\varvec{Y}}_{c-pseudo}-\text {O}_1\right) ,$$

and

14 $${\widehat{\varvec{b}}}={\hat{D}}{\widehat{V_c}}^{-1}\left( {\varvec{Y}}_{c-pseudo}-\text {O}_1-{\varvec{U}}{\widehat{\varvec{\gamma}}}\right) .$$

The updated estimates of the variance parameters, $\lambda$ and $\sigma$ are obtained by a Newton–Raphson iterative procedure as follows:

15 $$\begin{aligned} \left( \begin{array}{c} {\widehat{\sigma}}\\ {\widehat{\lambda}} \end{array} \right) ^{updated}=\left( \begin{array}{c} {\widehat{\sigma}}\\ {\widehat{\lambda}} \end{array} \right) ^{old}+{\varvec{I}}^{-1}{\varvec{S}}. \end{aligned}$$

where ${\varvec{S}}$ is the score vector and ${\varvec{I}}$ is the expected information matrix based on REML likelihood for ${\varvec{Y}}_{c-pseudo}$. The expression for the elements of the score vector and information matrix, letting ${\varvec{\theta }}=(\theta _1, \theta _2)=(\sigma ,\lambda )$ are given by

$$\begin{aligned}&S_i = \frac{1}{2}\left( {\varvec{Y}}_{c-pseudo}-{\varvec{U}}{\widehat{{\varvec{\gamma }}}}-\text {O}_1\right) ^{\mathrm{T}}P \frac{\delta V_c}{\delta \theta _i} P \left( {\varvec{Y}}_{c-pseudo}-{\varvec{U}}{\widehat{{\varvec{\gamma }}}}-\text {O}_1\right) -\frac{1}{2} tr\left( P \frac{\delta V_c}{\delta \theta _i} \right) \end{aligned}$$

and

$$I_{jk}=-\frac{1}{2} tr\left( P \frac{\delta V_c}{\delta \theta _j} P \frac{\delta V_c}{\delta \theta _k} \right) ,$$

where $P= V_c^{-1}-V_c^{-1}{\varvec{U}}({\varvec{U}}^{\mathrm{T}}V_c^{-1}{\varvec{U}})^{-1}{\varvec{U}}^{\mathrm{T}}V_c^{-1}$. The derivatives of $V_c$ with respect to $\sigma$ and $\lambda$ are given below:

$$\begin{aligned} \frac{\delta V_c}{\delta \sigma }&= {} 2 \sigma {\varvec{R}}^{-1}_{\lambda }\\ \frac{\delta V_c}{\delta \lambda }&= {} \sigma ^2 {\varvec{R}}^{-1}_{\lambda }({\varvec{R}}-\varvec{I}){\varvec{R}}^{-1}_{\lambda }, \end{aligned}$$

where ${\varvec{R}}_{\lambda }=(1-\lambda )\varvec{I}+\lambda {\varvec{R}}$ and ${\varvec{R}}$ is the intrinsic autoregression matrix.

Repeated iteration of Eqs. (11)–(15) allow us to obtain reliable estimates of the region specific fixed effect and random effect parameters. Convergence is achieved when the change in parameter estimates are less than a prespecified tolerance level (<1*e*−3, in the simulation study reported). Approximate standard errors for $\lambda$ and $\sigma$ are obtained from the above information matrix in the usual way.

## Appendix 2

See Table 7.

**Table 7:** Application of indiCAR with age as a continuous predictor

Regression coefficients	Estimates	SE
Intercept	−1.493	0.047
Age	−0.027	0.001
Sex		
Female	Ref.	
Male	−0.043	0.020
Year of diagnosis		
2001	Ref.	
2002	0.021	0.038
2003	0.083	0.038
2004	0.019	0.038
2005	0.095	0.037
2006	0.038	0.038
2007	−0.022	0.038
2008	−0.004	0.038
2009	−0.315	0.042
ARIA		
Major cities	Ref.	
Inner regional Australia	−0.006	0.022
Outer regional Australia	−0.118	0.037
Remote or very remote Australia	−0.192	0.142
Cancer type		
Breast cancer	Ref.	
Lung cancer	0.240	0.037
Colon and rectum cancer	−0.463	0.040
Haematological malignancy	1.497	0.029
Other cancer	−0.986	0.031
No. of major comorbidities		
0	Ref.	
1	0.413	0.026
2	0.682	0.026
3	0.589	0.036
4+	0.594	0.035
SEIFA		
Most disadvantaged	Ref.	
2	−0.089	0.043
3	−0.078	0.038
4	−0.134	0.045
Least disadvantaged	−0.144	0.053
Variance parameter		
*σ*	0.209	0.022
*λ*	0.992	0.012

## Abbreviations

CAR: Conditional Auto-Regressive
indiCAR: individual level covariate adjusted conditional auto-regressive model
SEIFA: Socio-Economic Index For Areas
ARIA: Accessibility/Remoteness Index of Australia
NSW: New South Wales
